# Establishing and Facilitating Large-Scale Manuscript Collaborations via Social Media: Novel Method and Tools for Replication

**DOI:** 10.2196/25077

**Published:** 2021-05-17

**Authors:** Kimberly D Acquaviva

**Affiliations:** 1 School of Nursing University of Virginia Charlottesville, VA United States

**Keywords:** social media, crowdsourcing, collaboration, health professions, medicine, scholarship, literature, research

## Abstract

**Background:**

Authorship teams in the health professions are typically composed of scholars who are acquainted with one another before a manuscript is written. Even if a scholar has identified a diverse group of collaborators outside their usual network, writing an article with a large number of co-authors poses significant logistical challenges.

**Objective:**

This paper describes a novel method for establishing and facilitating large-scale manuscript collaborations via social media.

**Methods:**

On September 11, 2020, I used the social media platform Twitter to invite people to collaborate on an article I had drafted. Anyone who wanted to collaborate was welcome, regardless of discipline, specialty, title, country of residence, or degree completion. During the 25 days that followed, I used Google Docs, Google Sheets, and Google Forms to manage all aspects of the collaboration.

**Results:**

The collaboration resulted in the completion of 2 manuscripts in a 25-day period. The International Council of Medical Journal Editors authorship criteria were met by 40 collaborators for the first article (“Documenting Social Media Engagement as Scholarship: A New Model for Assessing Academic Accomplishment for the Health Professions”) and 35 collaborators for the second article (“The Benefits of Using Social Media as a Health Professional in Academia”). The authorship teams for both articles were notably diverse, with 17%-18% (7/40 and 6/35, respectively) of authors identifying as a person of color and/or underrepresented minority, 37%-38% (15/40 and 13/35, respectively) identifying as LGBTQ+ (lesbian, gay, bisexual, transgender, gender non-conforming, queer and/or questioning), 73%-74% (29/40 and 26/35, respectively) using she/her pronouns, and 20%-23% (9/40 and 7/35, respectively) identifying as a person with a disability.

**Conclusions:**

Scholars in the health professions can use this paper in conjunction with the tools provided to replicate this process in carrying out their own large-scale manuscript collaborations.

## Introduction

In the health professions, we typically collaborate with people we already know or are at least acquainted with. There is a practical reason for this: We have to know someone exists in order to think of them as a potential collaborator. However, this can lead to authorship teams that lack diversity. Even if a scholar has identified a diverse group of collaborators outside their usual network, writing an article with a large number of co-authors can be a logistical nightmare. This paper describes a novel method for establishing and facilitating large-scale manuscript collaborations via social media.

## Methods

### Finding Collaborators

On September 11, 2020, I posted a series of tweets (a “thread”) on the social media platform Twitter, inviting people to collaborate with me on an article I had drafted [1]. Textbox 1 shows the transcript of the tweets in the thread.

The first tweet in the thread was shared with the >7400 people who follow me on Twitter; 2330 people engaged with the first tweet in the thread: 1991 people viewed the details about this tweet, 161 people clicked on my profile, 122 people clicked a heart icon to indicate they “liked” the tweet, 29 people replied to the tweet, and 27 people shared the tweet with their followers by retweeting it.

Less than 24 hours after I posted the thread inviting people to collaborate with me, 31 people had entered their names and affiliations onto the title page of the Google Doc to indicate their desire to serve as co-authors [2].

**Transcript of the tweets in the thread.**

Publication opportunity for MD, NP, and RN tweeps! I'm writing a piece on how to document social media engagement as public scholarship on CVs and dossiers in medicine and nursing. Target Journal = @AcadMedJournal There's no widely-accepted format for how to do this so I thought it was about time we fixed that. :) Since the article is about social media engagement, I decided to take a risk and use social media to recruit co-authors and add to/revise the draft manuscript. Academic Medicine uses the ICMJE definition of authorship: “Authorship is based on (1) substantial contributions to conception and design, or acquisition of data, or analysis and interpretation of data, (2) drafting the article or revising it critically for ”...important intellectual content, (3) final approval of the version to be published, and (4) agreement to be accountable for all aspects of the work in ensuring that questions related to the accuracy or integrity of any part of the work are appropriately “...investigated and resolved. Authors must meet conditions 1, 2, 3, and 4.” If you're willing to meet conditions 1, 2, 3, and 4, AND you're willing to turn around your edits in the next 7 days -- a timeframe I picked solely because I want to see how fast we can actually do this -- here's how you can co-author this paper with me. Step 1. Go to the Google Doc and type in your name and affiliation on the title page in a blue highlighted spots (you can add more spots if you need to). ** Rest assured your name will NOT be included on the submission unless you give approval of the final version.** Step 2. Make the article better. Work your magic. Don't insert comments -- add to or revise the actual text of the article. You have good ideas -- your ideas will make this article much better! Next Friday I'll take whatever edits have been made and will create a final version of the submission. I'll email it to everyone who listed their name on the title page. If you're happy with the final version and you meet the authorship criteria, bingo - you're a co-author. For a control-freak like me this is absolutely terrifying, BTW. So, here's the link to the Google Doc: [link to original Google Doc]. It's a rough draft, y'all -- like, ROUGH. If we pull this off, we can change the way CVs and dossiers look at med schools and nursing schools. The last person who did that was Ernest Boyer 30 years ago. How cool is that? In terms of the co-authors, I *really* want to have a diverse group. We can't have a bunch of white, cis, heterosexual, able-bodied folx writing this. If you're white, cis, heterosexual, and able-bodied, I'd still love for you to be a coauthor. Just saying that I'm hoping for a diverse group overall. I'm excited to see what comes of this. Thanks for being open to being part of this adventure! Here's the link to the Google Doc if you missed it earlier: [link to original Google Doc]. Thanks everybody!”

### Coordinating the Collaboration

To manage the large number of potential co-authors, I created a Google Sheet and posted a link to it at the top of the title page of the Google Doc containing the draft article. With the link to the Google Sheet, I included a note encouraging co-authors to indicate in the Google Sheet whether they identified as someone who is LGBTQ+ (lesbian, gay, bisexual, transgender, gender non-conforming, queer and/or questioning), a person of color/under-represented minority, and/or a person with a disability because this would be helpful info for us to have collectively so that we have a sense of the diversity of our authorship team (I have created a blank version of the Co-Author Info Sheet for anyone interested in replicating the process described in this innovation report) [3]. There was no screening process for who could or could not join in the collaboration. Anyone who wanted to collaborate was welcome, regardless of discipline, specialty, title, country of residence, or degree completion.

Over the next 7 days, the article grew and changed considerably. Students, residents, fellows, and more senior scholars joined as collaborators, and no contributions to the article were viewed as more or less important based on the status of the collaborator making the contribution. Collaborators made edits to the article and inserted comments or questions that were then answered by other collaborators, occasionally in real time when multiple people were working on the document simultaneously. By September 18, 2020 — my original target date for completing the article — it was clear the article had expanded to have 2 foci rather than 1. I pulled content from Article #1 into a second Google Doc to build an outline for Article #2, then shared the link to the outline with all collaborators.

To keep things simple throughout the collaboration process, the first page of the original Google Doc was where I posted messages to the collaborators as well as links to the revised Article #1 (guidelines for documenting social media contributions) and what had become a draft of Article #2 (the benefits of social media for health professionals in academia). This approach preserved the comments made by collaborators on the original draft so that we could maintain a history of everyone’s contributions.

### Ensuring the Integrity of Authorship Designation

With such a large team of potential co-authors, I recognized that I would need to build several checkpoints into the collaboration process to ensure that individuals who met the International Council of Medical Journal Editors (ICMJE) criteria for authorship would be credited as such on the manuscript. The ICMJE defines authorship as follows: “The ICMJE recommends that authorship be based on the following 4 criteria: 1. Substantial contributions to the conception or design of the work; or the acquisition, analysis, or interpretation of data for the work; AND 2. Drafting the work or revising it critically for important intellectual content; AND 3. Final approval of the version to be published; AND 4. Agreement to be accountable for all aspects of the work in ensuring that questions related to the accuracy or integrity of any part of the work are appropriately investigated and resolved. In addition to being accountable for the parts of the work he or she has done, an author should be able to identify which co-authors are responsible for specific other parts of the work. In addition, authors should have confidence in the integrity of the contributions of their co-authors. All those designated as authors should meet all four criteria for authorship, and all who meet the four criteria should be identified as authors. Those who do not meet all four criteria should be acknowledged” [4].

To address this, I asked co-authors to describe their specific contributions to each manuscript in a Google Sheet; then, I sent them a “Co-Author Attestation Form” at 2 different points in the manuscript preparation process. This form asked them to attest to having met all 4 of the ICMJE authorship criteria. All members of the authorship team were able to see one another’s contributions to the manuscript in the Google Doc as well as their descriptions of those contributions in the Google Sheet, so there was ample opportunity for concerns to be raised if an individual was not pulling their weight as a co-author. No such concerns were raised. In a few cases, individuals recognized during the co-author attestation checkpoints that they themselves did not meet the ICMJE authorship criteria, and as such, they requested being moved to the acknowledgements. Overall, this process seems to have been both efficient and effective.

### Revising, Rewriting, and Negotiating

On September 18, 2020, one collaborator (J Mugele) took the lead in completely rewriting Article #1 to improve flow and cohesion. This collaborator’s contributions were substantial in reshaping the way in which Article #1 was conceptualized, so I asked him to serve as second author. Although none of the co-authors expressed concerns about this, in hindsight, I should have asked the other collaborators if they supported this decision. At the time, I was struggling to manage what had grown into a team of 45 active, engaged collaborators; Mugele’s leadership in revising the article was a life buoy, and I grabbed it. Mugele sent me the revised article on September 19, 2020, and I posted it to the rest of the collaborators later that day as a link from the original Google Doc. I asked all collaborators to make final edits by Friday, September 25, 2020 and asked them to indicate their preference in the Google Sheet for listing co-authors after the first 2 authors (Acquaviva and Mugele).

Between September 19, 2020 and September 25, 2020, I followed up with collaborators via Twitter direct message to remind them to finish entering their info on the Google Sheet. The vast majority of collaborators indicated a preference for listing co-authors after Acquaviva and Mugele in alphabetical order, so I edited the title page of the manuscript accordingly. On September 25, 2020, when the article was finalized and all edits had been received, I put together 2 different Google Forms to send to collaborators. I emailed “Co-Author Attestation v. 1” to collaborators who had already documented in the Google Sheet that they met the first 2 ICMJE authorship criteria. The form asked them to indicate they approved the final version of the article and met the last 2 ICMJE authorship criteria. I emailed “Co-Author Attestation v. 2” to collaborators who had not yet documented in the Google Sheet that they met the first 2 ICMJE authorship criteria. The form asked them to describe how they met the first criteria and then indicate whether they met the other 3 criteria. I have created blank versions of the Co-Author Attestation v.1 [5] and Co-Author Attestation v.2 [6] for readers who are interested in replicating the approach described in this innovation report. The last Co-Author Attestation form for Article #1 was received on September 29, 2020. In keeping with the practice I follow with all co-authored manuscripts I write, I ran the manuscript through iThenticate prior to submission to ensure that it was free of plagiarism. None was found.

## Article #2

**Figure 1:**
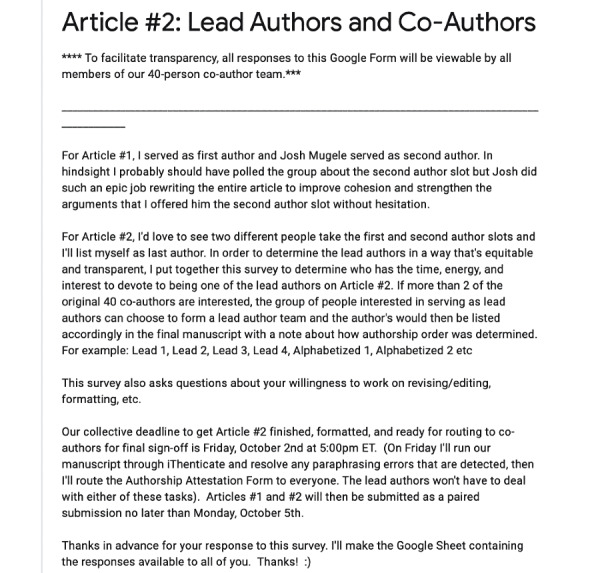
Message contained within the authorship interest form.

With Article #1 finalized, I then began the process of moving Article #2 toward completion. On September 27, 2020, I sent a Google Form to the 40 original co-authors and one individual who received an acknowledgement in Article #1 but made substantial contributions to the draft of Article #2. Because all 41 of these individuals had made significant contributions to the draft of Article #2, I wanted to give each person in the group the opportunity to take a lead role on the manuscript. The Google Form contained the message shown in Figure 1.

**Figure 2:**
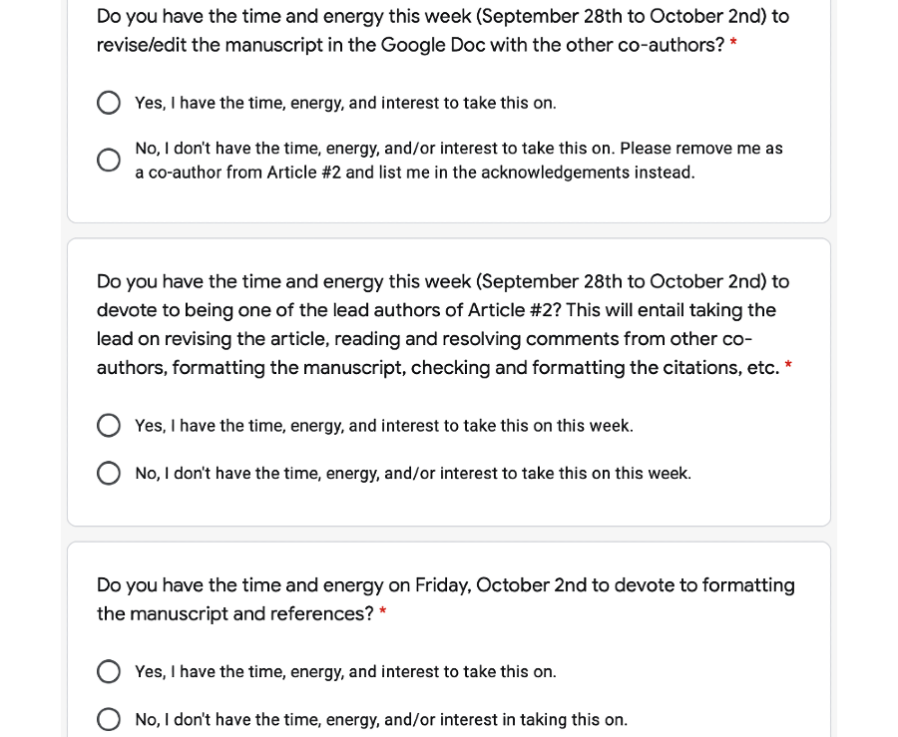
Questions on the authorship interest form.

The Google Form contained 3 questions (Figure 2).

**Figure 3:**
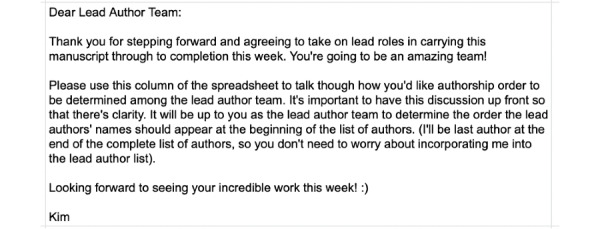
Message to the lead author team.

As responses to the Google Form were received, they automatically fed into a Google Sheet that was viewable by all members of the 41-person team. I created additional tabs in the Google Sheet labeled “Lead Author Team,” “Revising/Editing Team,” “Formatting Team,” and “Move to Acknowledgements” and then copied individual responses into these sheets. On the evening of September 27, 2020, I posted the message shown in Figure 3 as both a comment and a cell in the Lead Author Team sheet.

Over the next 7 days, the members of the Lead Author Team and Co-Author Team made considerable revisions to the manuscript. On October 3, 2020, I posted a link at the top of the manuscript Google Doc to a Google Form. The Google Form asked members of the authorship team if they happened to know the reference for 2 sentences that had been entered into the draft, as well as asked them about their contributions to the manuscript and their preferences for author order if they were a member of the Lead Author Team. Responses to the Google Form quickly yielded answers to the 2 reference questions but did little to answer the question of what order the lead author team members should be listed. On October 4, 2020, I asked a member of the Lead Author Team (Christopher Carroll) if he would take the lead on communicating with the rest of the lead author team via email to reach consensus on author order. Within 12 hours, the members of the Lead Author Team had reached consensus on the order their names should be listed on the final manuscript. Without exception, members of the Lead Author Team were generous in recognizing the contributions of other members and humble in conveying their own.

On October 5, 2020, I repeated the authorship attestation process followed with Article #1. I emailed the Co-Author Attestation Google Form along with a link to the final manuscript to all 39 collaborators. The last Co-Author Attestation form for Article #2 was received on October 5, 2020. In keeping with the practice I follow with all co-authored manuscripts I write, I ran the manuscript through iThenticate prior to submission to ensure that it was free of plagiarism. None was found.

## Results

### Article #1: Documenting Social Media Engagement as Scholarship: New Model for Assessing Academic Accomplishment for the Health Professions [7]

Of the 45 collaborators who had originally entered their names into the title page of the Google Doc as potential co-authors, 40 collaborators ended up meeting the ICMJE authorship criteria, 4 collaborators decided that their contributions were more appropriate for recognition in an acknowledgement, and 1 collaborator dropped out because they were unable to contribute. The authorship team was notably diverse (Table 1).

**Table 1:** Authorship team composition for article #1.

Article #1	Identifies as a person of color and/or an under-represented minority, n (%)	Identifies as LGBTQ+^a^, n (%)	Uses she/her pronouns	Identifies as a person with a disability
Authorship team (40 people)	7 (18)	15 (38)	29 (73)	9 (23)

^a^LGBTQ+: lesbian, gay, bisexual, transgender, gender non-conforming, queer and/or questioning.

To view the diverse array of degrees, licenses, and certifications held by the authorship team, you can view the article online [7].

After submitting the manuscript to Academic Medicine, we received a rejection within a matter of days. No feedback was provided. The authorship team revised the manuscript for submission to the Journal of Medical Internet Research (JMIR) and opted for open peer review. We received a revise-and-resubmit decision and then revised the manuscript to address the reviewers’ comments and suggestions. The manuscript was then accepted for publication and published in December 2020. The day the article was published online, one of the members of the authorship team noticed that their name was not listed on the article: Apparently, I had made an error during the process of entering the metadata into the journal’s online submission system. I was mortified. I immediately owned up to the error publicly on Twitter and contacted the authorship team to alert them directly. Because a correction to the list of authors in a journal article requires the approval of every member of the authorship team, I routed the corrigendum to every author for their review and signature. The error was corrected in the list of authors within a matter of days.

### Article #2: The Benefits of Using Social Media as a Health Professional in Academia

Of the 41 collaborators from Article #1 who were surveyed at the beginning of the Article #2 revision process to determine their interest in serving as a lead author or co-author on the second manuscript, 2 individuals asked to be moved to the acknowledgements section of Article #2. Of the remaining 39 collaborators, 7 indicated interest in serving on the lead author team. When the manuscript was formatted and finalized and the Authorship Attestation Form was routed on October 3, 2020, 35 collaborators ended up meeting the ICMJE authorship criteria, and 4 collaborators agreed to be moved to the acknowledgements section. The authorship team for Article #2 was as diverse as Article #1, with marked diversity among the Lead Author Team in particular (Table 2).

**Table 2:** Authorship team composition for article #2.

Article #2	Identifies as a person of color and/or an under-represented minority, n (%)	Identifies as LGBTQ+^a^, n (%)	Uses she/her pronouns, n (%)	Identifies as a person with a disability, n (%)
Lead authorship team (7 people)	2 (29)	3 (43)	3 (43)	2 (29)
Authorship team (35 people)	6 (17)	13 (37)	26 (74)	7 (20)

^a^LGBTQ+: lesbian, gay, bisexual, transgender, gender non-conforming, queer and/or questioning.

After submitting the manuscript to Academic Medicine, we received a rejection accompanied by helpful feedback for revising the paper for submission to another journal. The overarching theme running through the reviewers’ comments was that we needed to focus the article more narrowly — a valid criticism with which the members of the authorship team agreed. The authorship team is in the process of revising the manuscript to address the issues raised by the Academic Medicine reviewers. Because Academic Medicine did not invite us to resubmit a revised manuscript, we plan to submit the paper to another journal that is better aligned with the subject matter.

### Relationship Between the Three Manuscripts

Congruent with the ICMJE’s “Recommendations for the Conduct, Reporting, Editing, and Publication of Scholarly Work in Medical Journals” [4], this manuscript is not “…reporting work that has already been reported in large part in a published article or is contained in or closely related to another paper that has been submitted or accepted for publication elsewhere.” This manuscript is narrowly focused on describing the method used to establish and facilitate a large-scale collaboration using social media. The manuscript titled “Documenting Social Media Engagement as Scholarship: A New Model for Assessing Academic Accomplishment for the Health Professions” [7] presents the guidelines created through the process described in this manuscript, while the manuscript titled “The Benefits of Using Social Media as a Health Professional in Academia” (unpublished) examines the benefits of social media engagement.

### Conclusion

This paper and the 2 manuscripts described therein are evidence that it is possible to establish and facilitate large-scale manuscript collaborations via social media. Open collaboration on manuscripts in the health professions is one way to ensure diverse authorship teams and facilitate collaboration across disciplines. Scholars can use this innovation report in conjunction with the tools provided to replicate this process in carrying out their own large-scale manuscript collaborations.

## Abbreviations

ICMJE: International Council of Medical Journal Editors
LGBTQ+: lesbian, gay, bisexual, transgender, gender non-conforming, queer and/or questioning

## References

[1] Acquaviva K (2020). Thread by @kimacquaviva. Thread Reader App.

[2] Acquaviva K (2020). Tweet: It's only been 21 hrs since. Twitter.

[3] Acquaviva K (2020). Co-Author Info Sheet. Google Sheets.

[4] (2019). Recommendations for the Conduct, Reporting, Editing and Publication of Scholarly Work in Medical Journals. International Committee of Medical Journal Editors (ICMJE).

[5] Acquaviva K (2020). Co-Author Attestation v1. Google Forms.

[6] Acquaviva K (2020). Co-Author Attestation v2. Google Forms.

[7] Acquaviva KD, Mugele J, Abadilla N, Adamson T, Bernstein SL, Bhayani RK, Büchi AE, Burbage D, Carroll CL, Davis SP, Dhawan N, Eaton A, English K, Grier JT, Gurney MK, Hahn ES, Haq H, Huang B, Jain S, Jun J, Kerr WT, Keyes T, Kirby AR, Leary M, Marr M, Major A, Meisel JV, Petersen EA, Raguan B, Rhodes A, Rupert DD, Sam-Agudu NA, Saul N, Shah JR, Sheldon LK, Sinclair CT, Spencer K, Strand NH, Streed Jr Cg, Trudell AM (2020). Documenting Social Media Engagement as Scholarship: A New Model for Assessing Academic Accomplishment for the Health Professions. J Med Internet Res.

